# Supplementary material for: Next-Generation Sequencing of Four Mitochondrial Genomes of Dolichovespula (Hymenoptera: Vespidae) with a Phylogenetic Analysis and Divergence Time Estimation of Vespidae
Source: Animals (Basel). 2022 Nov 1;12(21):3004. doi: 10.3390/ani12213004 (PMC9657509; doi:10.3390/ani12213004)
Supplement: Supplementary file 1 [file animals-12-03004-s001.zip › Supplementary Figures.pdf]

Supplementary Figures

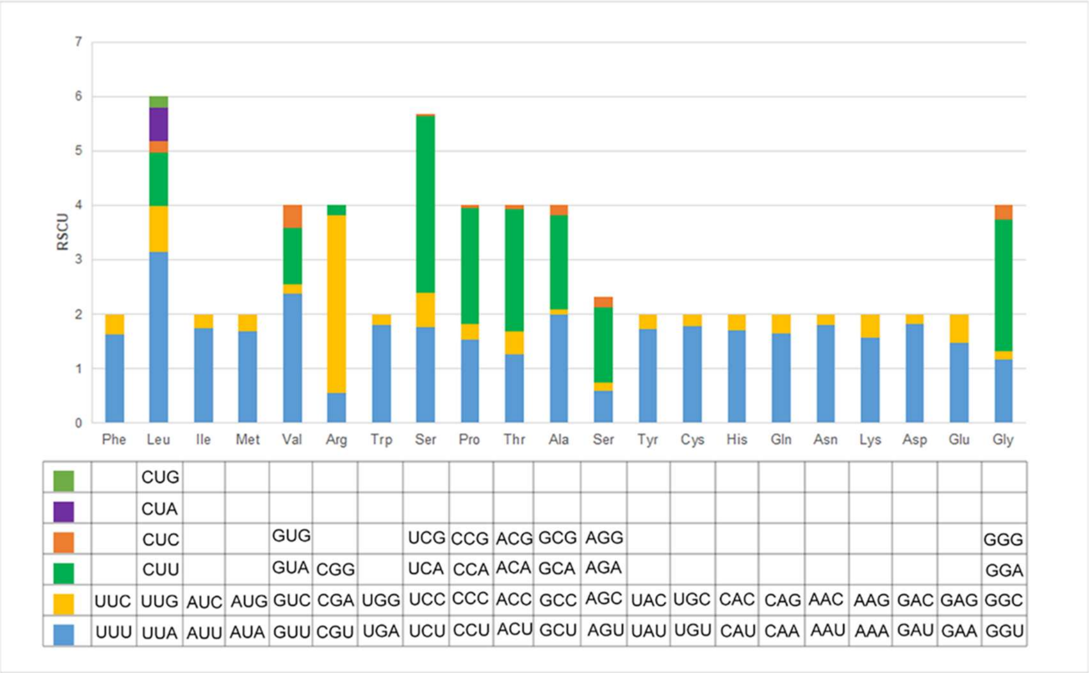

Figure S1. Relative synonymous codon usage (RSCU) for PCGs of the mitochondrial genomes of *Dolichovespula flora.*

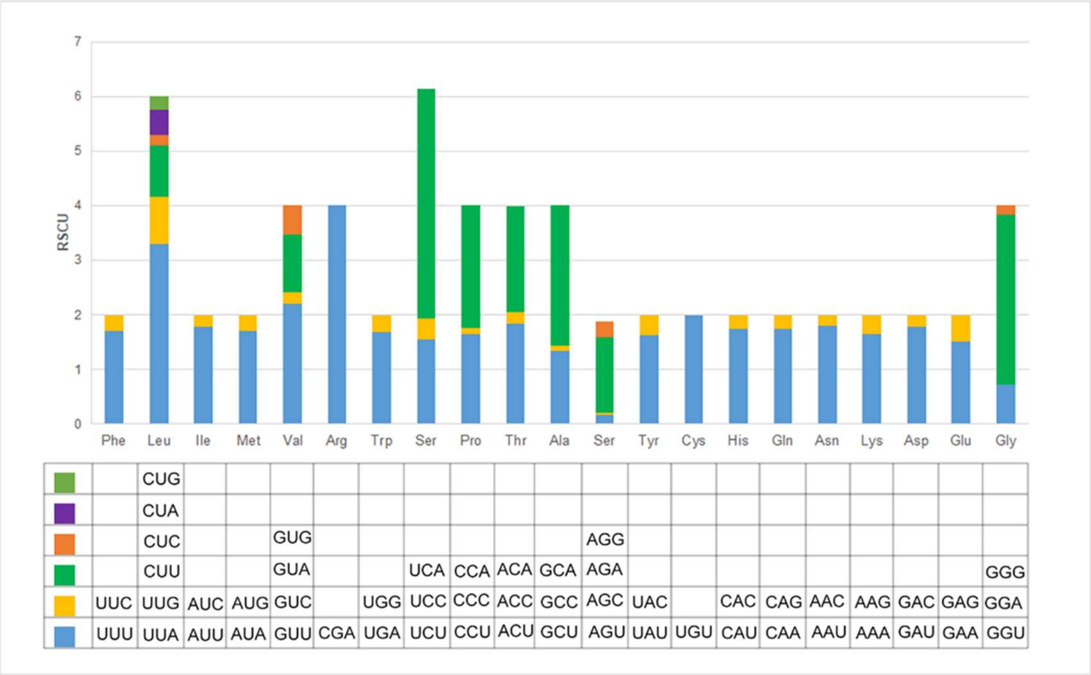

Figure S2. Relative synonymous codon usage (RSCU) for PCGs of the mitochondrial genomes of *Dolichovespula lama.*

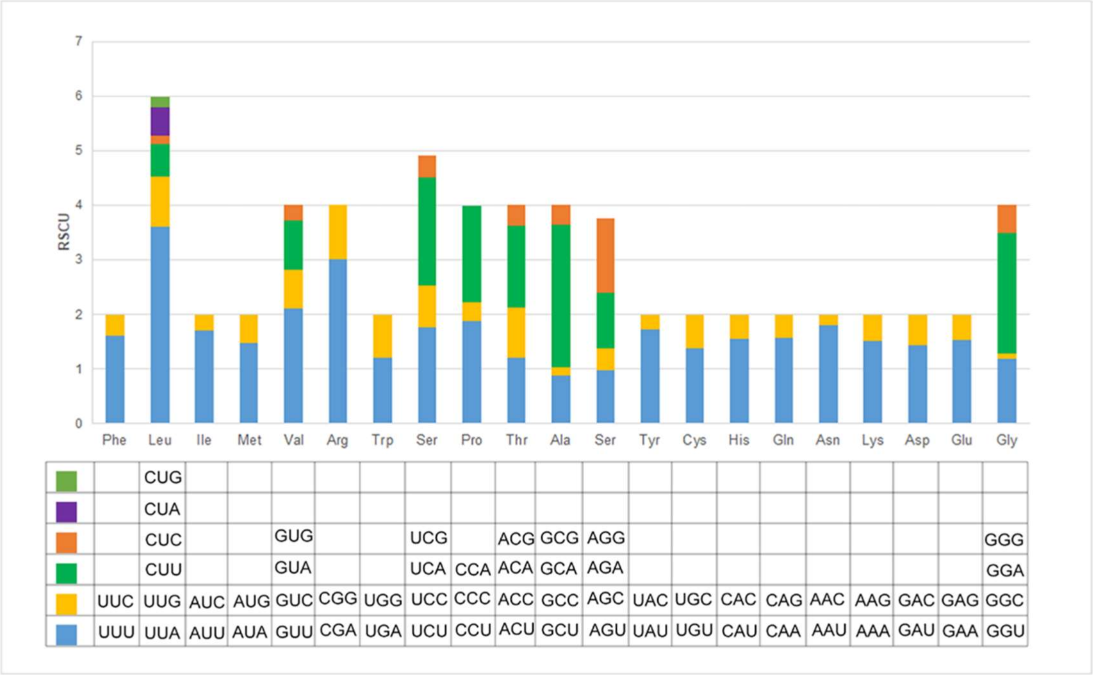

Figure S3. Relative synonymous codon usage (RSCU) for PCGs of the mitochondrial genomes of *Dolichovespula saxonica.*

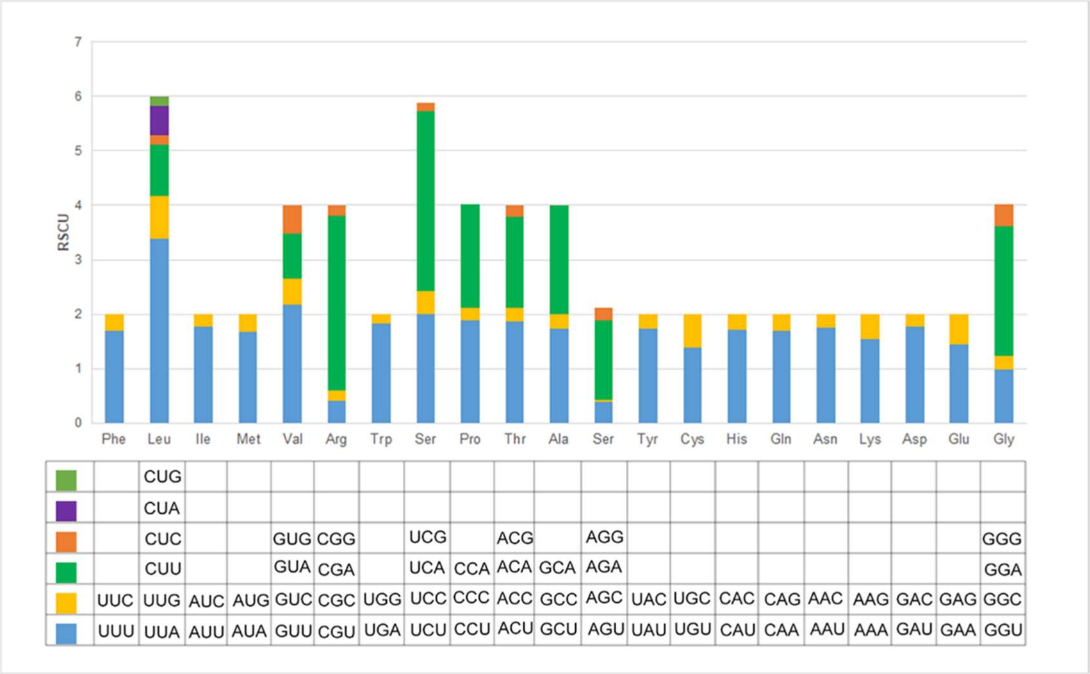

Figure S4. Relative synonymous codon usage (RSCU) for PCGs of the mitochondrial genomes of *Dolichovespula xanthicincta.*

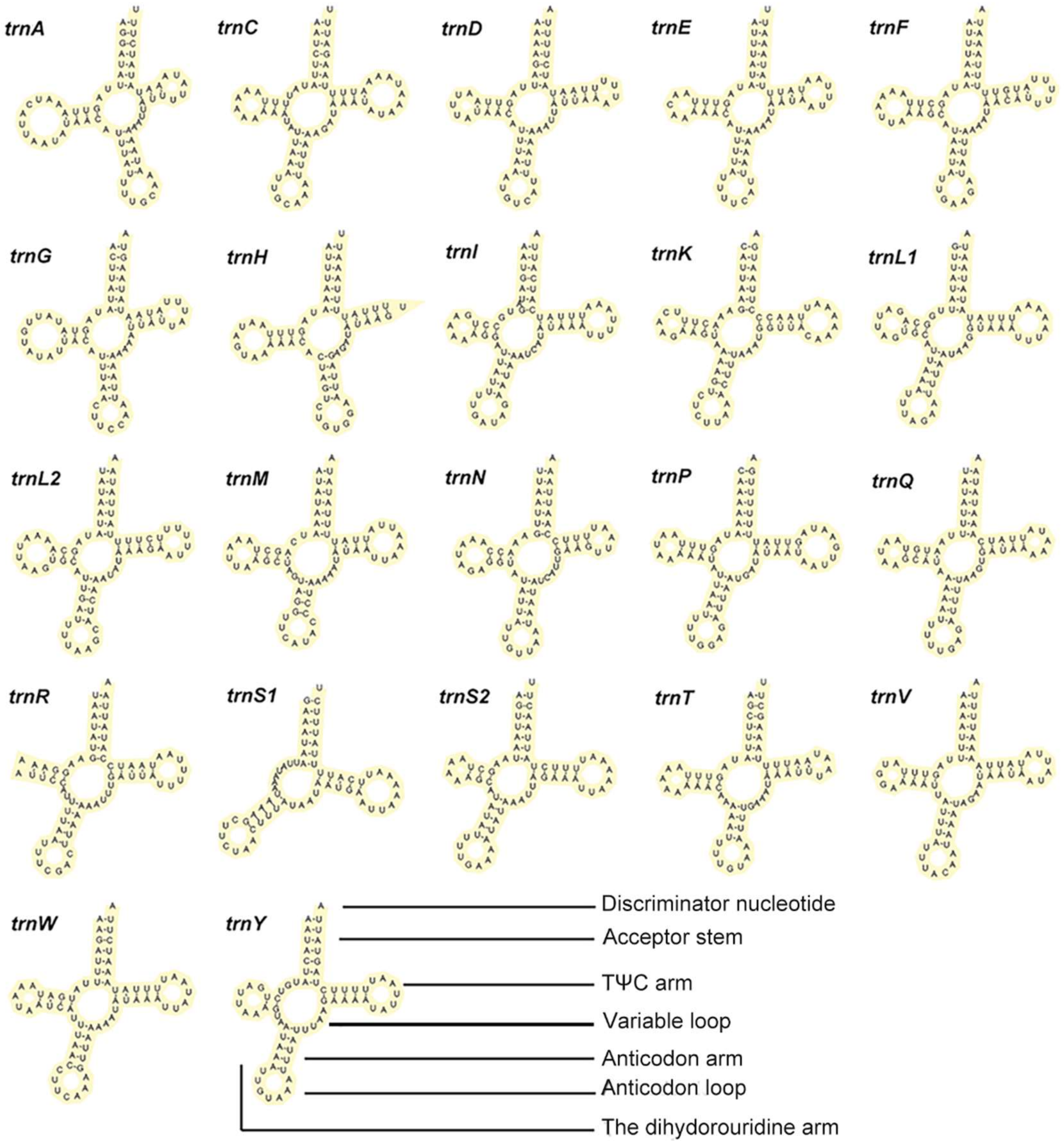

Figure S5. Cloverleaf structure of 22 tRNAs in the mitochondrial genome of *Dolichovespula flora*.

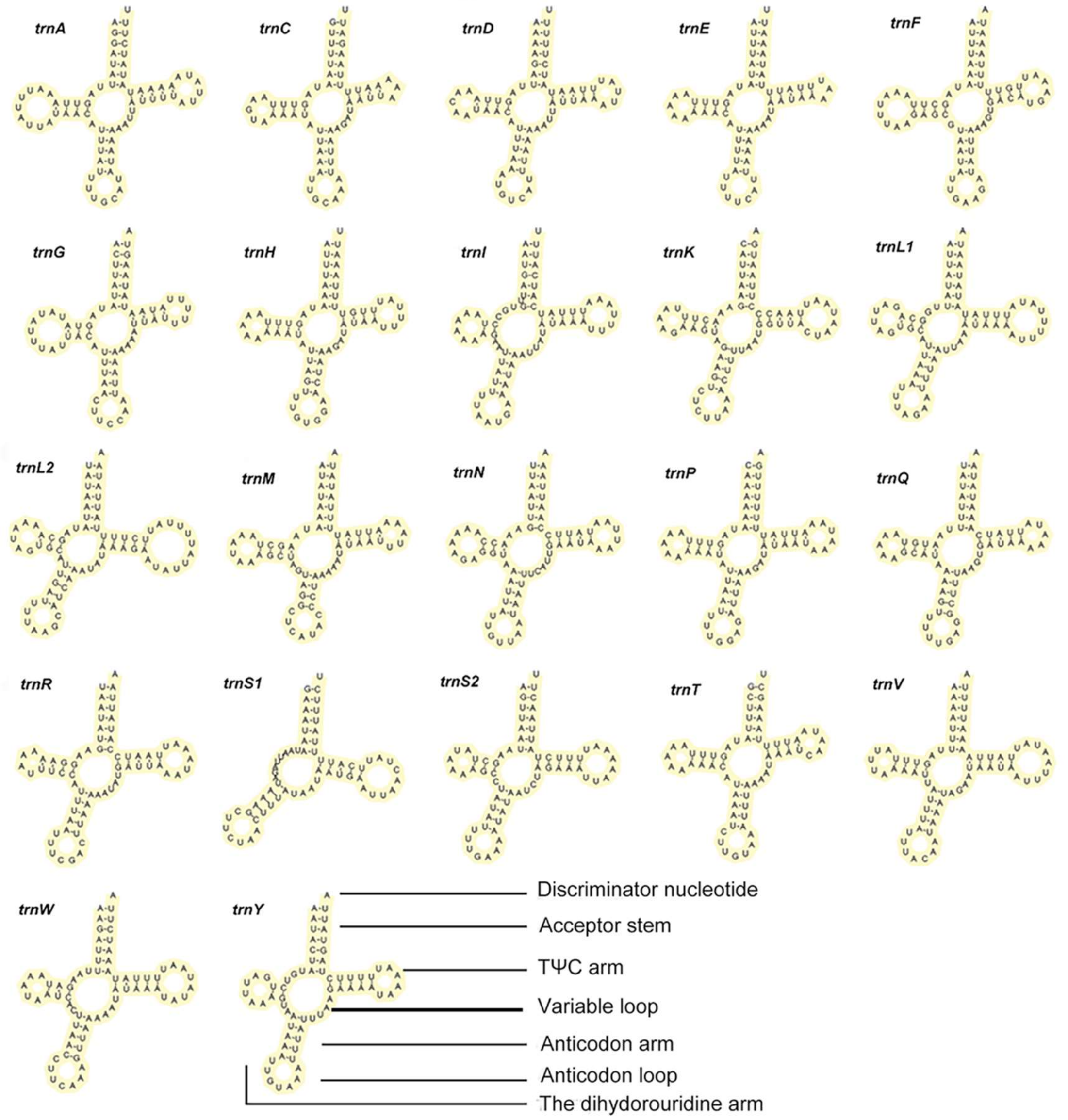

Figure S6. Cloverleaf structure of 22 tRNAs in the mitochondrial genome of *Dolichovespula lama*.

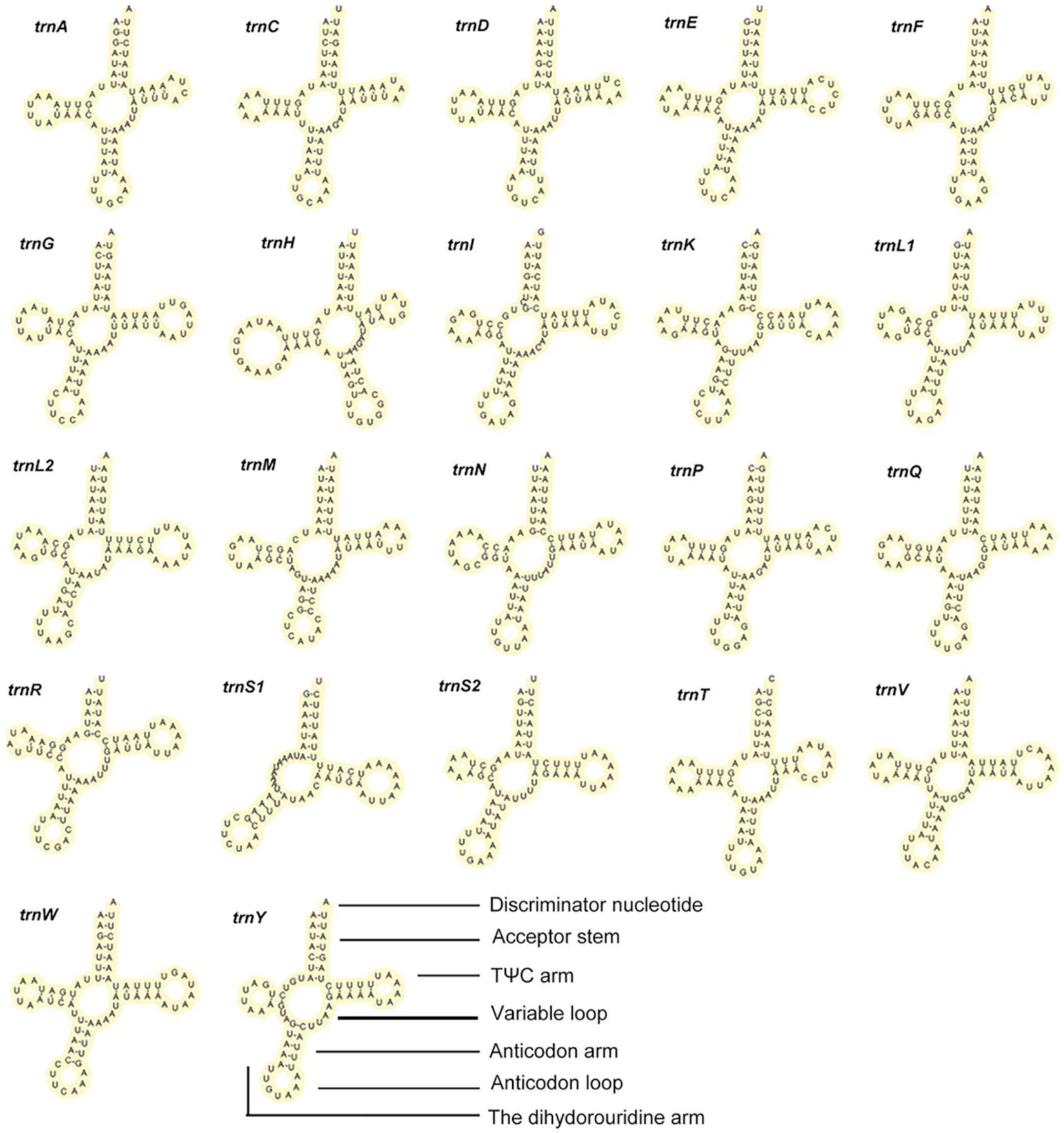

Figure S7. Cloverleaf structure of 22 tRNAs in the mitochondrial genome of *Dolichovespula saxonica*.

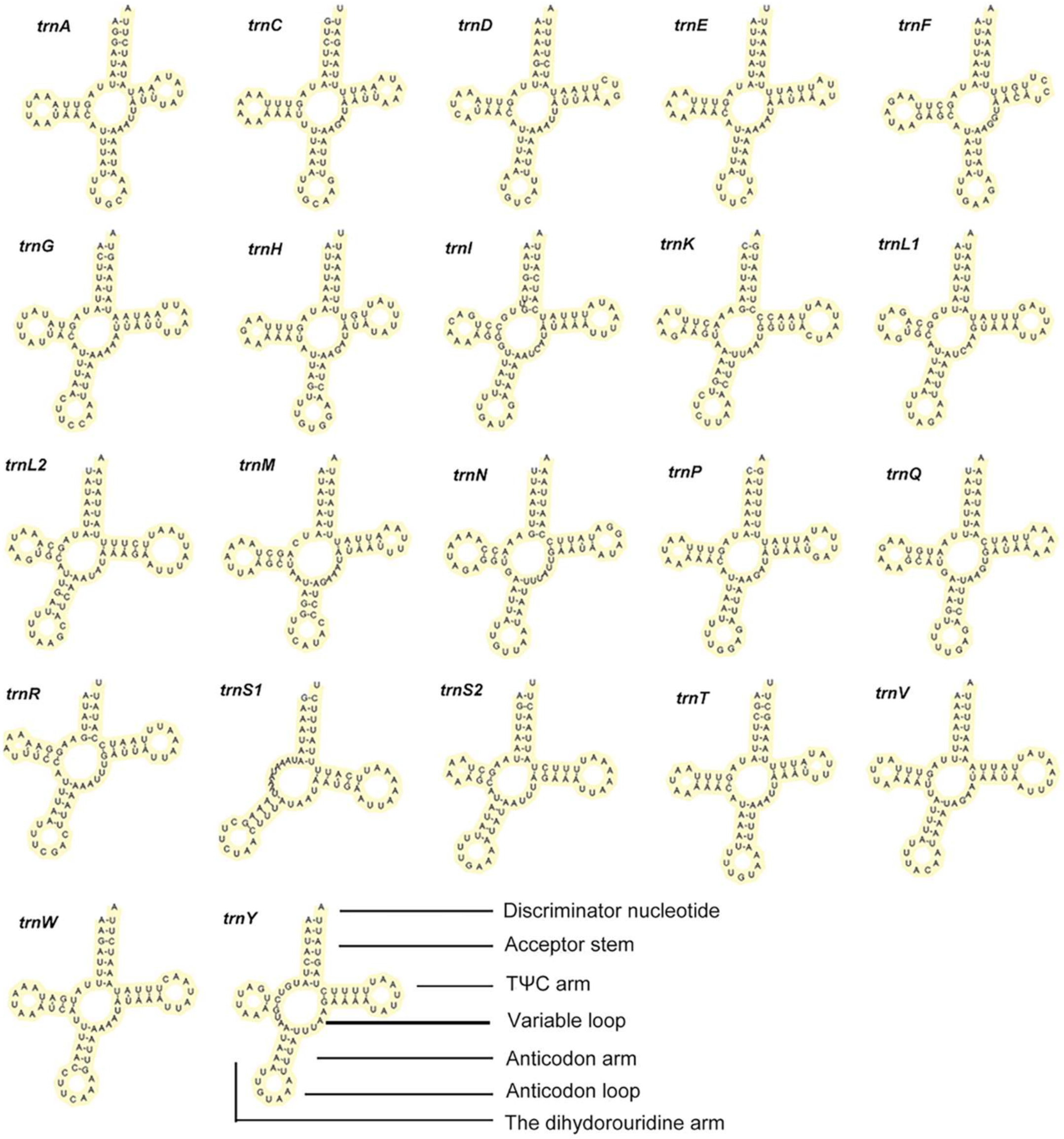

Figure S8. Cloverleaf structure of 22 tRNAs in the mitochondrial genome of *Dolichovespula xanthicincta*.

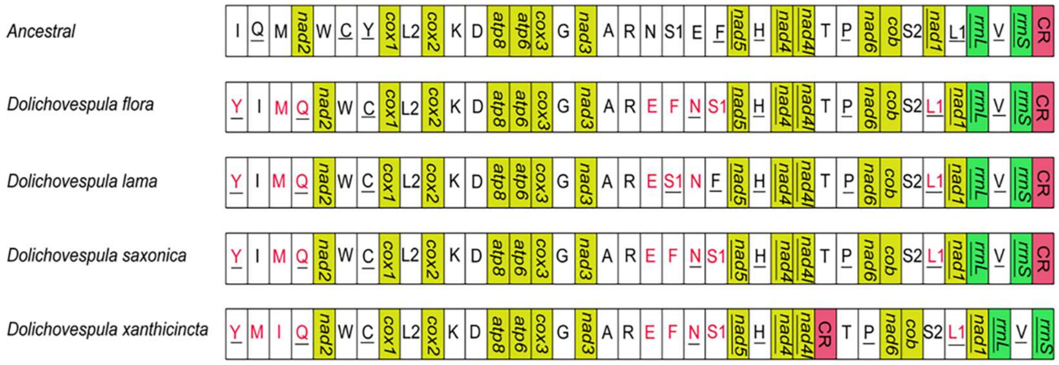

Figure S9. Mitochondrial genome organization of Vespidae referenced with the ancestral insect mitochondrial genomes. The underlined symbols are located on the N-

strand and others on the J-strand. The white, green, yellow and pink blocks denote tRNAs, rRNAs, PCGs and control regions, respectively. The red font means rearranged genes.
