# Supplementary material for: Next-Generation Sequencing of Four Mitochondrial Genomes of Dolichovespula (Hymenoptera: Vespidae) with a Phylogenetic Analysis and Divergence Time Estimation of Vespidae
Source: Animals (Basel). 2022 Nov 1;12(21):3004. doi: 10.3390/ani12213004 (PMC9657509; doi:10.3390/ani12213004)
Supplement: Supplementary file 1 [file animals-12-03004-s001.zip › Supplementary Schemes.pdf]

Table S1. Collection information of *Dolichovespula* newly sequenced in this study.

Species	Time	Locality	Collector
D. flora	19.vii.2013	Mt. Shaohua, Huaxian, Shaanxi, China	Jiangli Tan
D. lama	19.vii.2013	Yadong, Tibet, China	Tao Li
D. saxonica	3.viii.1983	LinJiang, Jilin, China	Baozhen Hua, ZL Wu
D. xanthicincta	8.vii.2013	Chayu, Tibet, China	Tao Li

Table S2. Mitochondrial genomes of Vespidae in GenBank.

Family	Subfamily	Species	Genomes size (bp)	Gene number	Accession Number
Vespidae	Vespinae	*Dolichovespula flora*	16481	37	**OP250139**
Vespidae	Vespinae	*Dolichovespula lama*	16065	37	**OP250140**
Vespidae	Vespinae	*Dolichovespula panda*	17137	37	**KY293679**
Vespidae	Vespinae	*Dolichovespula saxonica*	16420	37	**OP250141**
Vespidae	Vespinae	*Dolichovespula xanthicincta*	15965	37	**OP250142**
Vespidae	Vespinae	*Vespula germanica*	16342	33	**KR703583**
Vespidae	Vespinae	*Vespula flaviceps*	17489	37	**MK248830**
Vespidae	Vespinae	*Vespula vulgaris*	18184	33	**MK737077**
			17971	33	**MK737082**
			17999	33	**MK737081**
			18141	33	**MK737080**
			17980	33	**MK737079**
			17981	33	**MK737078**
			17996	33	**MK737083**
			18133	33	**MK737076**
			18147	33	**MK737075**
			17975	33	**MK737074**
Vespidae	Vespinae	*Vespa mandarinia*	16878	37	**LC541728**
			16864	37	**LC541730**
			15902	37	**KR059904**
			16872	37	**LC541729**
			16864	37	**LC541727**
Vespidae	Vespinae	*Vespa affinis*	19109	37	**AP018371**
Vespidae	Vespinae	*Vespa velutina nigrithorax*	16475	37	**KY091645**
Vespidae	Vespinae	*Vespa velutina*	16388	37	**AP018484**
			16388	37	**AP018483**
			16416	37	**AP017943**
Vespidae	Vespinae	*Vespa simillima simillima*	18340	37	**MN542756**
Vespidae	Vespinae	*Vespa simillima xanthoptera*	17885	37	**MN833127**
Vespidae	Vespinae	*Vespa basalis*	16735	37	**MK440075**
Vespidae	Vespinae	*Vespa ducalis*	15779	37	**KX950825**
Vespidae	Vespinae	*Vespa orientalis*	16101	37	**KY563657**
Vespidae	Vespinae	*Vespa bicolor*	16937	35	**KJ735511**

Vespidae	Polistinae	Vespidae sp.MT 2014	16278	36	**KM244667**
Vespidae	Polistinae	*Polistes riparius*	16383	37	**LC519884**
Vespidae	Polistinae	*Polistes jokahamae*	16616	34	**KR052468**
Vespidae	Polistinae	*Polistes* sp.MD1	14741	34	**EU024653**
Vespidae	Polistinae	*Parapolybia crocea*	16619	37	**KY679828**
Vespidae	Polistinae	*Parapolybia indica*	16824	37	**MH065757**
Vespidae	Polistinae	*Parapolybia nodosa*	19621	37	**MH065756**
Vespidae	Polistinae	*Parapolybia flava*	15709	37	**MH065755**
Vespidae	Polistinae	*Parapolybia tinctipennis*	16673	37	**MH065754**
Vespidae	Polistinae	*Parapolybia varia*	18096	37	**MH065752**
			17360	37	**MH065753**
			16564	37	**MH065751**
Vespidae	Eumeninae	*Abispa ephippium*	16953	41	**EU302588**
Vespidae	Eumeninae	*Orancistrocerus aterrimus*	17972	38	**KY941926**
Vespidae	Eumeninae	*Rhynchium aff. brunneum* GX	23122	38	**MK051032**
Vespidae	Eumeninae	*Rhynchium aff. brunneum* YN	23251	38	**MK051031**
Vespidae	Eumeninae	*Antodynerus aff. limbatus* YN	17975	38	**MK051025**
Vespidae	Eumeninae	*Antodynerus aff. limbatus* XZ	15742	36	**MK051024**
Vespidae	Eumeninae	*Antodynerus aff. limbatus* GX	16111	36	**MK051023**
Vespidae	Eumeninae	*Allorhynchium* sp. GX	19646	38	**MK051021**
Vespidae	Eumeninae	*Allorhynchium* sp. YN	19198	38	**MK051022**
Vespidae	Eumeninae	*Anterhynchium abdominale*	16488	36	**MK051029**
Vespidae	Eumeninae	*Anterhynchium (Dirhynchium)* sp. QHZ-2020	16512	36	**MK051028**
Vespidae	Eumeninae	*Anterhynchium aff. flavomarginatum* SC	15934	35	**MK051027**
Vespidae	Eumeninae	*Anterhynchium aff. flavomarginatum* HB	15196	35	**MK051026**
Vespidae	Eumeninae	*Rhynchium quinquecinctum*	16317	36	**MK051030**
Vespidae	Stenogastrinae	*Eustenogaster scitula*	17867	37	**KY856830**
Formicidae	Mymicinae	*Solenopsis richteri*	15560	37	**HQ215539**
Formicidae	Mymicinae	*Myrmica scabrinodis*	15310	37	**LN607806**

Note: Black indicates the mitochondrial genome data used in the phylogenetic analysis. Orange indicates the data have not been selected in our phylogenetic analysis.

Table S3. *Dolichovespula* and outgroup species included in our study, and corresponding Genbank accession numbers.

**Species**	*cob*	*cox1*	*cox2*	*rrnS*	*rrnL*
Dolichovespula omissa	KT257131	KT257111	KT257121	KT225584	KT250517
Dolichovespula adulterina	KT257129	KT257109	KT257119	KT225582	MN823246
Dolichovespula arctica	KT257130	KT257110	KT257120	KT225583	KT250518
Dolichovespula maculata	KJ147287	KJ147231	KJ147259	-	KJ147202

Dolichovespula media	KJ147288	KJ147232	KJ147260	KJ147176	KJ147203
Dolichovespula sylvestris	KJ147291	KJ147235	KJ147263	KJ147179	KJ147206
Dolichovespula pacifica	KJ147289	KJ147233	KJ147261	KJ147177	KJ147204
Dolichovespula albida	KJ147285	KJ147229	KJ147257	KJ147200	KJ147227
Dolichovespula arenaria	KJ147286	KJ147230	KJ147258	KJ147175	KJ147228
Dolichovespula panda	KY293679	KY293679	KY293679	KY293679	KY293679
Dolichovespula flora	OP250139	OP250139	OP250139	OP250139	OP250139
Dolichovespula lama	OP250140	OP250140	OP250140	OP250140	OP250140
Dolichovespula saxonica	OP250141	OP250141	OP250141	OP250141	OP250141
Dolichovespula xanthicincta	OP250142	OP250142	OP250142	OP250142	OP250142
Polistes riparius	LC519884	LC519884	LC519884	LC519884	LC519884
Polistes jokahamae	KR052468	KR052468	KR052468	KR052468	KR052468

Table S4. The best partitioning scheme selected by PartitionFinder for BI analysis in Vespidae.

Datasets	Subset Partitions	Best Models
PCG123<br><br>29 partitions	P1 (atp6_pos1)	GTR+I+G
	P2 (atp6_pos2, nad3_pos2)	GTR+I+G
	P3 (atp6_pos3, cob_pos2, cox3_pos2)	GTR+I+G
	P4 (atp8_pos1, atp8_pos3, nad2_pos2, nad6_pos2)	GTR+I+G
	P5 (atp8_pos2)	HKY+I+G
	P6 (cob_pos1)	GTR+I+G
	P7 (cob_pos3)	GTR+I+G
	P8 (cox1_pos1)	GTR+I+G
	P9 (cox1_pos2)	GTR+I
	P10 (cox1_pos3)	GTR+G
	P11 (cox2_pos1)	GTR+G
	P12 (cox2_pos2)	GTR+G
	P13 (cox2_pos3, nad3_pos1)	GTR+G
	P14 (cox3_pos1)	GTR+I+G
	P15 (cox3_pos3)	GTR+G
	P16 (nad1_pos1)	GTR+I+G
	P17 (nad1_pos2, nad5_pos1)	GTR+I+G
	P18 (nad1_pos3)	GTR+I+G
	P19 (nad2_pos1, nad6_pos3)	GTR+I+G

	P20 (nad2_pos3)	GTR+G
	P21 (nad3_pos3)	GTR+G
	P22 (nad4_pos1, nad5_pos3)	GTR+I+G
	P23 (nad4_pos2)	GTR+I+G
	P24 (nad4_pos3)	GTR+G
	P25 (nad4l_pos1)	GTR+G
	P26 (nad4l_pos2)	GTR+G
	P27 (nad4l_pos3)	HKY+G
	P28 (nad5_pos2)	GTR+I+G
	P29 (nad6_pos1)	GTR+I+G
PCG123rRNAs<br><br>31 partitions	P1 (atp6_pos1)	GTR+I+G
	P2 (atp6_pos2, nad3_pos2)	GTR+I+G
	P3 (atp6_pos3, cob_pos2, cox3_pos2)	GTR+I+G
	P4 (atp8_pos3, nad2_pos2, nad6_pos2, atp8_pos1)	GTR+I+G
	P5 (atp8_pos2)	HKY+I+G
	P6 (cob_pos1)	GTR+I+G
	P7 (cob_pos3)	GTR+I+G
	P8 (cox1_pos1)	GTR+I+G
	P9 (cox1_pos2)	GTR+I
	P10 (cox1_pos3)	GTR+G
	P11 (cox2_pos1)	GTR+G
	P12 (cox2_pos2)	GTR+G
	P13 (nad3_pos1, cox2_pos3)	GTR+G
	P14 (cox3_pos1)	GTR+I+G
	P15 (cox3_pos3)	GTR+G
	P16 (nad1_pos1)	GTR+I+G
	P17 (nad1_pos2, nad5_pos1)	GTR+I+G
	P18 (nad1_pos3)	GTR+I+G
	P19 (nad2_pos1, nad6_pos3)	GTR+I+G
	P20 (nad2_pos3)	GTR+G
	P21 (nad3_pos3)	GTR+G

	P22 (nad4_pos1, nad5_pos3)	GTR+I+G
	P23 (nad4_pos2)	GTR+I+G
	P24 (nad4_pos3)	GTR+G
	P25 (nad4l_pos1)	GTR+G
	P26 (nad4l_pos2)	GTR+G
	P27 (nad4l_pos3)	HKY+G
	P28 (nad5_pos2)	GTR+I+G
	P29 (nad6_pos1)	GTR+I+G
	P30 (16Srrn)	GTR+I+G
	P31 (12Srrn)	GTR+G

Table S5. The best partitioning scheme selected by PartitionFinder for ML analysis in Vespidae.

Datasets	Subset Partitions	Best Models
PCG123	P1 (atp6_pos1)	GTR+I+G
	P2 (nad3_pos2, atp6_pos2)	GTR+I+G
29 partitions	P3 (atp6_pos3, cox3_pos2, cob_pos2)	GTR+I+G
	P4 (nad2_pos2, atp8_pos3, nad6_pos2, atp8_pos1)	GTR+I+G
	P5 (atp8_pos2)	GTR+G
	P6 (cob_pos1)	GTR+I+G
	P7 (cob_pos3)	GTR+I+G
	P8 (cox1_pos1)	GTR+I+G
	P9 (cox1_pos2)	GTR+G
	P10 (cox1_pos3)	GTR+G
	P11 (cox2_pos1)	GTR+G
	P12 (cox2_pos2)	GTR+G
	P13 (nad3_pos1, cox2_pos3)	GTR+G
	P14 (cox3_pos1)	GTR+I+G
	P15 (cox3_pos3)	GTR+I+G
	P16 (nad1_pos1)	GTR+I+G
	P17 (nad1_pos2, nad5_pos1)	GTR+I+G
	P18 (nad1_pos3)	GTR+I+G
	P19 (nad2_pos1, nad6_pos3)	GTR+I+G
	P20 (nad2_pos3)	GTR+G
	P21 (nad3_pos3)	GTR+G
	P22 (nad4_pos1, nad5_pos3)	GTR+I+G
	P23 (nad4_pos2)	GTR+I+G
	P24 (nad4_pos3)	GTR+I+G

	P25 (nad4l_pos1)	GTR+G
	P26 (nad4l_pos2)	GTR+I+G
	P27 (nad4l_pos3)	GTR+G
	P28 (nad5_pos2)	GTR+I+G
	P29 (nad6_pos1)	GTR+I+G
PCG123rRNAs<br><br>32 partitions	P1 (atp6_pos1)	GTR+I+G
	P2 (nad3_pos2, atp6_pos2)	GTR+I+G
	P3 (atp6_pos3, cob_pos2, cox3_pos2)	GTR+I+G
	P4 (atp8_pos3, nad6_pos2, atp8_pos1)	GTR+I+G
	P5 (atp8_pos2)	GTR+I+G
	P6 (cob_pos1)	GTR+I+G
	P7 (cob_pos3)	GTR+I+G
	P8 (cox1_pos1)	GTR+I+G
	P9 (cox1_pos2)	GTR+I+G
	P10 (cox1_pos3)	GTR+I+G
	P11 (cox2_pos1)	GTR+G
	P12 (cox2_pos2)	GTR+G
	P13 (nad3_pos1, cox2_pos3)	GTR+G
	P14 (cox3_pos1)	GTR+I+G
	P15 (cox3_pos3)	GTR+I+G
	P16 (nad1_pos1)	GTR+I+G
	P17 (nad5_pos1, nad1_pos2)	GTR+I+G
	P18 (nad1_pos3)	GTR+I+G
	P19 (nad6_pos3, nad2_pos1)	GTR+I+G
	P20 (nad2_pos2)	GTR+I+G
	P21 (nad2_pos3)	GTR+G
	P22 (nad3_pos3)	GTR+G
	P23 (nad4_pos1, nad5_pos3)	GTR+I+G
	P24 (nad4_pos2)	GTR+I+G
	P25 (nad4_pos3)	GTR+I+G
	P26 (nad4l_pos1)	GTR+G
	P27 (nad4l_pos2)	GTR+I+G
	P28 (nad4l_pos3)	GTR+G
	P29 (nad5_pos2)	GTR+I+G
	P30 (nad6_pos1)	GTR+I+G
	P31 (16Srrn)	GTR+I+G
	P32 (12Srrn)	GTR+G

Table S6. The best partitioning scheme selected by PartitionFinder for BI analysis in *Dolichovespula*.

Datasets	Subset Partitions	Best Models
PCG123R<br>8 partitions	P1 (cob_pos1)	GTR+G
	P2 (cox1_pos3, cob_pos2)	GTR+G

	P3 (cox1_pos2, cob_pos3, cox1_pos1)	GTR+G
	P4 (cox2_pos1)	GTR+G
	P5 (cox2_pos2)	HKY+I+G
	P6 (cox2_pos3)	HKY+G
	P7 (16Srrn)	GTR+I+G
	P8 (12Srrn)	GTR+I+G

Table S7. The best partitioning scheme selected by PartitionFinder for ML analysis in *Dolichovespula*.

Datasets	Subset Partitions	Best Models
PCG123R<br>8 partitions	P1 (cob_pos1)	TIM+G
	P2 (cox1_pos3, cob_pos2)	TVM+G
	P3 (cox1_pos2, cob_pos3, cox1_pos1)	GTR+G
	P4 (cox2_pos1)	TVM+G
	P5 (cox2_pos2)	TRN+I+G
	P6 (cox2_pos3)	TIM+G
	P7 (16Srrn)	TIM+I+G
	P8 (12Srrn)	TVM+I+G

Table S8. Annotation of mitochondrial genomes of four *Dolichovespula* species.

Dolichovespula flora						
Gene	Strand	Position	Length (bp)	Start codon	Stop codon	Intergenic nucleotides (bp)
trnY	N	1-68	68			13
trnI	J	82-150	69			76
trnM	J	227-295	69			40
trnQ	N	336-402	67			16
nad2	J	419-1087<br>1466-1468	672	ATT	TAA	7
estimated gaps		1088-1465	378			
trnW	J	1476-1544	69			-8
trnC	N	1537-1606	70			39
cox1	J	1646-3181	1536	ATG	TAA	26
trnL2	J	3208-3276	69			0
cox2	J	3277-3960	684	ATT	TAA	4
trnK	J	3965-4034	70			16
trnD	J	4051-4116	66			0
atp8	J	4117-4281	165	ATT	TAA	-7
atp6	J	4275-4940	666	ATG	TAA	-1
cox3	J	4940-5724	785	ATG	TA	-1
trnG	J	5724-5791	68			0
nad3	J	5792-6148	357	ATA	TAA	6
trnA	J	6155-6223	69			-1
trnR	J	6223-6290	68			3

trnE	J	6294-6359	66			19
trnF	J	6397-6447	69			14
trnN	N	6462-6527	66			20
trnS1	J	6548-6609	62			8
nad5	N	6618-8288	1671	ATA	TAA	0
trnH	N	8289-8354	66			-1
nad4	N	8354-9673	1320	ATG	TAA	-7
nad4l	N	9667-9960	294	ATT	TAA	51
trnT	J	10012-10077	66			-1
trnP	N	10077-10147	71			12
nad6	J	10160-10717<br>10775-10777	561	ATG	TAA	3
estimated gaps		10718-10774	57			
cob	J	10781-11920	1140	ATG	TAA	12
trnS2	J	11933-11999	67			51
trnL1	N	12051-12119	69			25
nad1	N	12145-13106	962	ATA	TA	46
rrnL	N	13153-14519	1367			62
trnV	N	14582-14649	68			1
rrnS	N	14651-15412	762			0
A + T-rich region		15413-16481	1069			

Dolichovespula lama

Gene	Strand	Position	Length (bp)	Start codon	Stop codon	Intergenic nucleotides (bp)
trnY	N	1-67	67			36
trnI	J	103-172	70			40
trnM	J	213-280	68			141
trnQ	N	422-488	67			43
nad2	J	532-1584	1053	ATT	TAA	1
trnW	J	1586-1655	70			-7
trnC	N	1649-1711	63			2
cox1	J	1714-3249	1536	ATG	TAA	6
trnL2	J	3256-3328	73			0
cox2	J	3329-4012	684	ATT	TAA	28
trnK	J	4041-4110	70			24
trnD	J	4135-4201	67			0
atp8	J	4202-4363	162	ATT	TAA	-7
atp6	J	4357-5022	666	ATG	TAA	2
cox3	J	5025-5809	785	ATG	TA	-1
trnG	J	5809-5875	67			0
nad3	J	5876-6232	357	ATA	TAA	23
trnA	J	6256-6327	72			-1
trnR	J	6327-6395	69			17
trnE	J	6413-6477	65			45

trnS1	N	6523-6585	63			11
trnN	J	6597-6665	69			224
trnF	N	6890-6958	69			0
nad5	N	6959-8638	1680	ATT	TAA	-3
trnH	N	8636-8701	66			19
nad4	N	8721-10040	1320	ATG	TAA	-7
nad4l	N	10034-10321	288	ATA	TAA	72
trnT	J	10394-10458	65			5
trnP	N	10464-10531	68			33
nad6	J	10565-11125<br>11180-11182	564	ATG	TAA	1
estimated gaps		11126-11179	54			
cob	J	11184-12323	1140	ATG	TAA	20
trnS2	J	12344-12411	68			-1
trnL1	N	12411-12480	70			170
nad1	N	12651-13619	969	ATA	TAA	46
rrnL	N	13666-15049	1384			64
trnV	N	15114-15184	71			2
rrnS	N	15187-15951	765			0
A + T-rich region		15952-16065	114			

Dolichovespula saxonica

Gene	Strand	Position	Length (bp)	Start codon	Stop codon	Intergenic nucleotides (bp)
trnY	N	1-67	67			12
trnI	J	80-149	70			23
trnM	J	173-240	68			24
trnQ	N	265-332	68			17
nad2	J	350-1411	1062	ATG	TAA	8
trnW	J	1420-1489	70			-7
trnC	N	1483-1547	65			10
cox1	J	1558-2403<br>3091-3092	848	ATG	TA	286
estimated gaps		2404-3090	687			
trnL2	J	3379-3448	73			0
cox2	J	3449-4132	684	ATT	TAA	20
trnK	J	4153-4222	70			12
trnD	J	4235-4301	67			0
atp8	J	4302-4460	162	ATT	TAA	-7
atp6	J	4454-5119	666	ATG	TAA	-1
cox3	J	5119-5903	785	ATG	TA	-1
trnG	J	5903-5972	70			0
nad3	J	5973-6329	357	ATT	TAA	17
trnA	J	6347-6411	65			0
trnR	J	6412-6476	65			4

trnE	J	6481-6549	69			4
trnF	J	6554-6620	67			8
trnN	N	6629-6698	70			13
trnS1	J	6712-6774	63			77
nad5	N	6852-8531	1683	ATT	TAA	-3
trnH	N	8532-8602	71			8
nad4	N	8611-9924	1314	ATG	TAA	-7
nad4l	N	9918-10211	294	ATA	TAA	203
trnT	J	10415-10481	67			-2
trnP	N	10480-10546	67			20
nad6	J	10567-11130<br>11182-11184	567	ATG	TAA	0
estimated gaps		11131-11181	51			
cob	J	11185-12324	1140	ATG	TAA	14
trnS2	J	12339-12405	67			0
trnL1	N	12406-12473	68			68
nad1	N	12542-13510	969	ATA	TAA	46
rrnL	N	13557-14927	1371			65
trnV	N	14993-15063	71			3
rrnS	N	15067-15827	761			0
A + T-rich region		15828-16420	593			

Dolichovespula xanthicincta

Gene	Strand	Position	Length (bp)	Start codon	Stop codon	Intergenic nucleotides (bp)
trnY	N	1-68	68			20
trnM	J	89-157	69			276
trnI	J	434-503	70			145
trnQ	N	649-716	68			42
nad2	J	759-1808	1050	ATT	TAA	8
trnW	J	1817-1886	70			-7
trnC	N	1880-1945	66			2
cox1	J	1948-3483	1536	ATG	TAA	3
trnL2	J	3487-3557	71			0
cox2	J	3558-4241	684	ATT	TAA	20
trnK	J	4262-4331	70			14
trnD	J	4346-4411	66			0
atp8	J	4412-4570	159	ATT	TAA	-7
atp6	J	4564-5229	666	ATG	TAA	-1
cox3	J	5229-6013	785	ATG	TA	-1
trnG	J	6013-6080	68			0
nad3	J	6081-6437	357	ATA	TAA	18
trnA	J	6456-6520	65			0
trnR	J	6521-6585	65			8
trnE	J	6594-6659	66			4

trnF	J	6664-6729	66			7
trnN	N	6737-6806	70			15
trnS1	J	6822-6884	63			28
nad5	N	6913-8589	1677	ATT	TAA	-3
trnH	N	8587-8657	71			6
nad4	N	8658-9971	1314	ATG	TAG	-7
nad4l	N	9965-10249	285	ATA	TAA	309
A + T-rich region		10250-10558	309			
trnT	J	10559-10623	65			7
trnP	N	10631-10698	68			5
nad6	J	10704-11294<br>11319-11321	594	ATG	TAA	0
estimated gaps		11295-11318	24			
cob	J	11322-12461	1140	ATG	TAA	9
trnS2	J	12471-12562	67			-1
trnL1	N	12537-12604	68			61
nad1	N	12666-13640	975	ATT	TAG	57
rrnL	N	13698-15058	1361			50
trnV	N	15109-15179	71			0
rrnS	N	15180-15965	786			0

Table S9. Codon usage in the mitochondrial genome of the four *Dolichovespula* species.

Dolichovespula flora				*Dolichovespula lama*				*Dolichovespula saxonica*				*Dolichovespula xanthicincta*			
AA	Codon	No.	RSCU	AA	Codon	No.	RSCU	AA	Codon	No.	RSCU	AA	Codon	No.	RSCU
Phe	UUU	372	1.62	Phe	UUU	387	1.7	Phe	UUU	414	1.6	Phe	UUU	378	1.69
	UUC	86	0.38		UUC	69	0.3		UUC	103	0.4		UUC	69	0.31
Leu	UUA	300	3.15	Leu	UUA	328	3.3	Leu	UUA	234	3.61	Leu	UUA	343	3.38
	UUG	80	0.84		UUG	85	0.86		UUG	59	0.91		UUG	81	0.8
	CUU	93	0.98		CUU	94	0.95		CUU	39	0.6		CUU	94	0.93
	CUC	19	0.2		CUC	19	0.19		CUC	10	0.15		CUC	17	0.17
	CUA	59	0.62		CUA	46	0.46		CUA	34	0.52		CUA	55	0.54
	CUG	21	0.22		CUG	24	0.24		CUG	13	0.2		CUG	18	0.18
Ile	AUU	317	1.75	Ile	AUU	331	1.78	Ile	AUU	349	1.7	Ile	AUU	332	1.77
	AUC	46	0.25		AUC	41	0.22		AUC	61	0.3		AUC	43	0.23
Met	AUA	185	1.68	Met	AUA	213	1.7	Met	AUA	134	1.48	Met	AUA	195	1.68
	AUG	35	0.32		AUG	38	0.3		AUG	47	0.52		AUG	37	0.32
Val	GUU	75	2.38	Val	GUU	85	2.21	Val	GUU	30	2.11	Val	GUU	71	2.17
	GUC	5	0.16		GUC	8	0.21		GUC	10	0.7		GUC	16	0.49
	GUA	33	1.05		GUA	40	1.04		GUA	13	0.91		GUA	27	0.82
	GUG	13	0.41		GUG	21	0.55		GUG	4	0.28		GUG	17	0.52
Arg	CGU	3	0.55	Arg	CGU	0	0	Arg	CGU	0	0	Arg	CGU	2	0.4
	CGC	0	0		CGC	0	0		CGC	0	0		CGC	1	0.2
	CGA	18	3.27		CGA	20	4		CGA	3	3		CGA	16	3.2
	CGG	1	0.18		CGG	0	0		CGG	1	1		CGG	1	0.2

Trp	UGA	67	1.79	Trp	UGA	69	1.68	Trp	UGA	59	1.2	Trp	UGA	61	1.82
	UGG	8	0.21		UGG	13	0.32		UGG	39	0.8		UGG	6	0.18
Ser	UCU	45	1.76	Ser	UCU	39	1.54	Ser	UCU	45	1.76	Ser	UCU	51	2
	UCC	16	0.63		UCC	10	0.4		UCC	31	0.77		UCC	11	0.43
	UCA	83	3.25		UCA	106	4.2		UCA	79	1.98		UCA	84	3.29
	UCG	1	0.04		UCG	0	0		UCG	16	0.4		UCG	4	0.16
Pro	CCU	26	1.53	Pro	CCU	28	1.65	Pro	CCU	16	1.88	Pro	CCU	33	1.89
	CCC	5	0.29		CCC	2	0.12		CCC	3	0.35		CCC	4	0.23
	CCA	36	2.12		CCA	38	2.24		CCA	15	1.76		CCA	33	1.89
	CCG	1	0.06		CCG	0	0		CCG	0	0		CCG	0	0
Thr	ACU	33	1.26	Thr	ACU	49	1.83	Thr	ACU	32	1.2	Thr	ACU	53	1.86
	ACC	11	0.42		ACC	6	0.22		ACC	25	0.93		ACC	7	0.25
	ACA	59	2.25		ACA	52	1.94		ACA	40	1.5		ACA	48	1.68
	ACG	2	0.08		ACG	0	0		ACG	10	0.37		ACG	6	0.21
Ala	GCU	22	2	Ala	GCU	14	1.33	Ala	GCU	5	0.87	Ala	GCU	20	1.74
	GCC	1	0.09		GCC	1	0.1		GCC	1	0.17		GCC	3	0.26
	GCA	19	1.73		GCA	27	2.57		GCA	15	2.61		GCA	23	2
	GCG	2	0.18		GCG	0	0		GCG	2	0.35		GCG	0	0
Ser	AGU	15	0.59	Ser	AGU	4	0.16	Ser	AGU	39	0.98	Ser	AGU	10	0.39
	AGC	4	0.16		AGC	1	0.04		AGC	16	0.4		AGC	1	0.04
	AGA	35	1.37		AGA	35	1.39		AGA	41	1.02		AGA	37	1.45
	AGG	5	0.2		AGG	7	0.28		AGG	54	1.35		AGG	6	0.24
Tyr	UAU	202	1.73	Tyr	UAU	211	1.62	Tyr	UAU	227	1.73	Tyr	UAU	221	1.74
	UAC	31	0.27		UAC	49	0.38		UAC	36	0.27		UAC	33	0.26
Cys	UGU	16	1.78	Cys	UGU	24	2	Cys	UGU	43	1.37	Cys	UGU	16	1.39
	UGC	2	0.22		UGC	0	0		UGC	20	0.63		UGC	7	0.61
His	CAU	83	1.71	His	CAU	92	1.75	His	CAU	48	1.55	His	CAU	90	1.71
	CAC	14	0.29		CAC	13	0.25		CAC	14	0.45		CAC	15	0.29
Gln	CAA	76	1.65	Gln	CAA	77	1.75	Gln	CAA	48	1.57	Gln	CAA	81	1.69
	CAG	16	0.35		CAG	11	0.25		CAG	13	0.43		CAG	15	0.31
Asn	AAU	147	1.8	Asn	AAU	155	1.79	Asn	AAU	339	1.8	Asn	AAU	152	1.76
	AAC	16	0.2		AAC	18	0.21		AAC	37	0.2		AAC	21	0.24
Lys	AAA	98	1.57	Lys	AAA	115	1.65	Lys	AAA	143	1.52	Lys	AAA	108	1.54
	AAG	27	0.43		AAG	24	0.35		AAG	45	0.48		AAG	32	0.46
Asp	GAU	78	1.81	Asp	GAU	80	1.78	Asp	GAU	38	1.43	Asp	GAU	86	1.77
	GAC	8	0.19		GAC	10	0.22		GAC	15	0.57		GAC	11	0.23
Glu	GAA	94	1.48	Glu	GAA	83	1.52	Glu	GAA	39	1.53	Glu	GAA	85	1.44
	GAG	33	0.52		GAG	26	0.48		GAG	12	0.47		GAG	33	0.56
Gly	GGU	22	1.16	Gly	GGU	13	0.72	Gly	GGU	14	1.19	Gly	GGU	20	0.99
	GGC	3	0.16		GGC	0	0		GGC	1	0.09		GGC	5	0.25
	GGA	46	2.42		GGA	56	3.11		GGA	26	2.21		GGA	48	2.37
	GGG	5	0.26		GGG	3	0.17		GGG	6	0.51		GGG	8	0.4
